# A clockmaker’s mathematics: a technology-based approach to the mathematical works of Jost Bürgi (1552–1632)

**DOI:** 10.1007/s00407-025-00347-7

**Published:** 2025-02-14

**Authors:** Damian Moosbrugger

**Affiliations:** Chair of History and Philosophy of Mathematics, Department of Humanities, Social and Political Sciences, Swiss Federal Institute of Technology Zurich (ETH Zürich), Clausiusstrasse 59, 8092 Zurich, Switzerland

## Abstract

In this article, I propose a new approach to analyze the interrelations between mathematics and technology. It has the potential to contribute methodologically to both the fields of history of mathematics as well as the study of computational technologies in the current context. Based on the conception of mathematics as a contingent human practice, I claim that the practical engagement with technology not only subjects new fields, materials, and problems to mathematical scrutiny but might even shape mathematics from within. To illustrate my approach and corroborate my thesis, I present a historical case study on the mathematical works of the Swiss clock- and instrument-maker Jost Bürgi (1552–1632). Besides being a practicing artisan, he left three mathematical treatises. The advancements in fine metal working at his time, exemplified in clockwork mechanisms and measuring instruments, not only motivated and directed Bürgi’s mathematical inquiries. Instead, I argue that the interaction with these technical apparatuses in practice has shaped the internal structure and workings of his mathematics, that is, its entities, justifications, presentations, proofs, and procedures. The close analysis of some aspects of his oeuvre, especially his notion(s) of the sine, his way of explaining the occurrence of multiple solutions in algebra, and his visual depiction of the bridging of ten in his logarithmic computational tool, reveals a potential integration of the experience and practical knowledge of a clockmaker into mathematics. I therefore make the point that his mathematical writings portray a clockmaker’s mathematics.

## Mathematics and the Hessen-Grossmann Thesis

The study of what has colloquially become known as the Scientific Revolution experienced a crucial shift in perspective in recent decades. Instead of focusing on the intellectual sphere, an increasing number of early modern science scholars draw our attention to the analysis of what was going on at the more practical level (e.g., Long 2011; Smith 2004; Valleriani 2017). In their approach, they managed to highlight the essential roles played by artisans and craftsmen in the transformations of the views, knowledge, and study of nature that occurred in sixteenth and seventeenth century Europe. As part of this trend, the cultures of mathematical practitioners at that time have become subject to scrutiny (e.g., Cormack et al. 2017; Morel 2022; Peters 2018). In this article, I wish to add another dimension to the interrelations between mathematics and technology.

A perspective on science that emphasizes its close connection to technology was expressed already in the 1930s. Boris Hessen (1931/2009; 1936/2022) showed that the development of theoretical mechanics in the seventeenth century must be understood as a response to the technical tasks faced in the economic spheres at that time. Similarly, Henryk Grossmann (1935/2009, 107) argued that

mechanistic philosophy and scientific mechanics derived their basic mechanical concepts from the observation of mechanisms, of *machines.*

Their combined views on the essential impact of technology on scientific thought have been generalized to the Hessen-Grossmann Thesis by Freudenthal and McLaughin (2009, 4):

Technology was developed *in order to* facilitate economic development and science developed *by means of* the study of the technology that was being applied or developed.

Science is thus conceptualized as emerging from technology by taking it as the subject matter of analysis, thereby not being reducible to it.

While it provides me with the basic underlying narrative for my endeavor, I propose to widen the framework of the Hessen–Grossmann Thesis to make it more fitting to my research. By integrating more recent perspectives on mathematics, science, and thought in general, this approach can offer new theoretical and methodological tools for the study of the interrelations between mathematics and technology. More specifically, returning to Grossmann’s claim quoted above as a starting point, I suggest an adaptation in three respects:

First, I argue that not only “mechanistic philosophy” or “scientific mechanics” was affected by the interaction with technology, but that this can be expected for the case of mathematics as well. Since the time of Hessen’s and Grossmann’s writings, a growing number of philosophers oppose viewing mathematics as a purely objective and absolute form of knowledge that is hence strictly cumulative (see e.g., Ernest 1998; Hersh 1997). The expanding current of history and philosophy of mathematical practice attests to this development in recent decades (e.g., Mancosu 2008; Robson and Stedall 2010; Sriraman 2024). Mathematics is seen as a contingent practical human endeavor, closely tied to the historically specific social interaction with material reality (see Asper 2008; Babu D. 2022). Within such a conception, the presence of “mechanisms”, “machines”, or—more fitting in the early modern context—mechanical devices such as clockworks or measuring instruments, beyond providing new objects to be studied mathematically, shapes the internal workings of mathematical reasoning.

Second, rather than on a contemplative category like “observation”, I propose to rely on the practical engagement with technology as a means of transmission of knowledge. Mental processes do not take place independently of our constant conjunction with material reality in practice (e.g., Malafouris 2013; Overmann 2019). Hence, the center of attention must be directed toward a figure I refer to as *technician-mathematician*. By this term, I wish to denote people that were involved in mathematical pursuits, that is, left some sort of mathematical record, while at the same time being engaged in technical practices such as the making of clocks or instruments.^1^ Technicians gained practical knowledge through the construction of artifacts, interaction with tools, and performance of measurements and calculations. The application of the technical experience of these practitioners in the field of mathematics comprises instances of production of knowledge by means of the merging of technological and mathematical practice.

Third, the devil lies in the detail. I will refrain from a scrutiny of such extensive occurrences as the emergence of the mechanistic world view and instead consider phenomena on a much smaller scale. More, specifically, I want to identify potential conceptual and procedural changes in the vernacular mathematical corpus of early modern technical practitioners with respect to the Latin university-based mathematics and attempt to trace them back to their professional occupations.

Changing the terms in Grossmann’s quote according to these three adaptations results in what can be seen as the general hypothesis I wish to defend: mathematics was shaped by the practical engagement with technology. More specifically, I suggest that the everyday interaction of technical practitioners—clockmakers, instrument-makers, master builders, etc.—with mechanical devices is reflected in their mathematical practice. Not only can it be expected to have motivated and guided their specific interests as mathematicians, thereby extending the range of topics, questions, and material subjected to mathematical scrutiny. I argue that it has contributed to changes within mathematics itself.

This paper is structured around a case study to substantiate the claim. It thereby illustrates my approach to analyze the interrelations between mathematics and technology, which offers an additional layer of explanation for the changes in early modern European science with the potential applications in other contexts as well. In what follows, I will thus present the mathematical oeuvre of the Renaissance clockmaker Jost Bürgi along the narrative offered by the Hessen–Grossmann Thesis. A special emphasis is put on three specific instances of his mathematics that reveal a potential origin in the technical apparatuses frequently used by him, that is, in clockwork mechanisms as well as the tools for their construction. The way in which he approached and dealt with problems of mathematics, I subsequently elaborate, corresponds to the experiential disposition of a trained artisan. Such a perspective, as will be shown, further strengthens the plausibility of assuming an interaction between technical and mathematical practice to have taken place in his case. Hence, I propose characterizing his mathematical work as a clockmaker’s mathematics.

## Meeting Jost Bürgi and the computational problem

An almost paradigmatic figure in the category of technician–mathematicians can be seen in the Swiss maker of clocks and instruments Jost Bürgi (1552–1632). Nowadays, he is mostly famous for the high-end planetary clocks and celestial globes he made. At the same time, there are three extant mathematical works written by him, which have been edited by Launert (2015), List and Bialas (1973), as well as Clark (2015). In this respect, Bürgi is best known for his invention of a logarithmic computational tool and—since its rather recent decryption—for his discovery of a unique method for computing sine values: his *artificium*.^2^ In this paper, I will focus on several distinctive features of his mathematics that have not yet received much attention.

Let me first offer a quick sketch of how it came to be that an artisan like Bürgi ended up as a part-time mathematician. Our protagonist was born into a family of craftsmen in Lichtensteig, a small town in the Toggenburg that lies in what is now the canton of St. Gallen in the east of Switzerland. His father as well as his grandfather had been locksmiths (Staudacher 2018, 27–29). Nothing is known with certainty about his youth. He probably first went through an apprenticeship as a locksmith, silver/goldsmith, or something similar, and was taught the craft of clockmaking later (55). The first historical record of him concerns his employment at the court of the landgrave of Hesse-Kassel as a princely clockmaker in 1579 (81–87).

Bürgi’s patron William IV is known for his interest in astronomy.^3^ In 1560, for instance, he set up a permanent observatory (Hamel 1998, 11). In this environment, Bürgi constructed the most precise observational clocks of his time. The verge escapement with foliot provided the basis for mechanical clocks up to then (e.g., Cipolla 1967, 57–58). Bürgi managed to reach a new level of consistency of spring-driven clocks by inventing and incorporating new mechanical elements, such as the remontoire or the cross-beat escapement, as well as by achieving perfection in his artisanship, for instance, using large finely made iron cogwheels (McNeil and Day 1996, 204–5; Staudacher 2018, 127–31). Moreover, he also developed more accurate instruments such as sextants and quadrants made from metal (Staudacher 2018, 92–96), participated in the determination of the positions of the fixed stars under the supervision of Christoph Rothmann and even made his own observations (Hamel 1998, 74).

Apart from measuring the sky, spherical astronomy consists of doing computations. To calculate the location of a planet from its angular distance to two known fixed stars, for instance, it was necessary to solve several instances of the spherical law of cosines (e.g., Van Brummelen 2013, 98)

$$ {\text{cos}}\left( c \right) = {\text{cos}}\left( a \right){\text{cos}}\left( b \right) + {\text{sin}}\left( a \right){\text{sin}}\left( b \right){\text{cos}}\left( C \right), $$

where *a*, *b*, and *c* denote the sides of the spherical triangle formed, e.g., by the planet and the two fixed stars on the celestial sphere, whereas *C* is the angle opposite of *c*. Since the numerical expressions of trigonometric entities often consist of many digits, astronomical calculation at that time, especially multiplication and division, was a tedious, time-consuming process, prone to errors. Further enhanced by the increasing mechanical precision, in practice, early modern astronomers therefore faced what I refer to as a *computational problem*: performing calculations used up a massive part of their active lives. As will be illustrated in the following, it can be assumed that Bürgi’s encounter with this technical issue motivated his mathematical inquiries.

## A proof of *prosthaphaeresis* via a new notion of the sine

The earliest known mathematical text by Bürgi is a manuscript titled *Fundamentum Astronomiae*.^4^ It was dedicated and presented to Rudolf II, the Emperor of the Holy Roman Empire and Bürgi’s future patron, in 1592 after handing over a celestial globe to him (Launert 2015, 6–8). Bürgi had probably already started working on the manuscript around the year 1586 (Staudacher 2018, 190). The treatise consists of two books. The second one covers the solution of all kinds of planar and spherical triangles, some of which are indeed used in astronomical calculations. However, it is a chapter of the first book to which we turn our attention.

After describing how to deal with sexagesimal numbers more generally, Bürgi commences the third chapter with the following statement:

From the effect of the numbers arises the invention of the *proportion*, which is found in a general and industrious way through the usual *operationem proportionis*, such as multiplication and division, or, however, in a much easier and nimbler way by *prosthaphaeresin*, that is, by addition and subtraction with the help of the sine (Launert 2015, 22).^5^

In other words, Bürgi tells us that there is a method to simplify multiplications or divisions by means of substituting them with additions or subtractions using the sine.^6^ What must have been on his mind is the computational problem of astronomy: How to perform the multiplications and divisions of many digits numbers faced in astronomical practice in a more efficient manner?

**Fig. 1:**
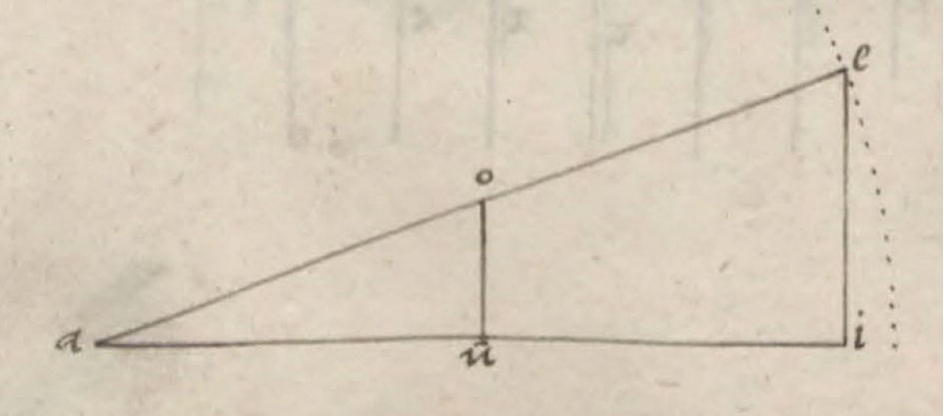
The representation of sine multiplication in Bürgi (1592): *Fundamentum Astronomiae*, Wroclaw University Library, Sig. IV Q 38a, fol. 17v

Here, I follow the reconstruction of Bürgi’s demonstration of the method that became known as *prosthaphaeresis* offered by Launert (2015, 22–29). It starts with a discussion of the triangle *aei* (Fig. 1). With reference to the second proposition of book six of Euclid’s *Elements*—which portrays a version of the intercept theorem—Bürgi argues that “the way the whole line *ae* relates to *ei*^7^ in the given triangle, so does the part *ao* to *ou*^8^” (22). Stated differently, according to Launert (24)

$$ ou = ei \times \frac{ao}{{ae}}. $$

It illustrates that the shift from *ei* to *ou* can be understood as the result of multiplication by a proportion. By identifying *ae* with the radius of a circle, he manages to interpret *ou* as the product of two sine values: *ei* is the sine of the angle *eai* and the proportion *ao*:*ae* can be treated as a sine value, too. Bürgi does not explicitly identify the representation with the multiplication of sines here, but the fact that he refers to multiplication as a “proportional operation” in the passage quoted above strongly suggests this reading.

**Fig. 2:**
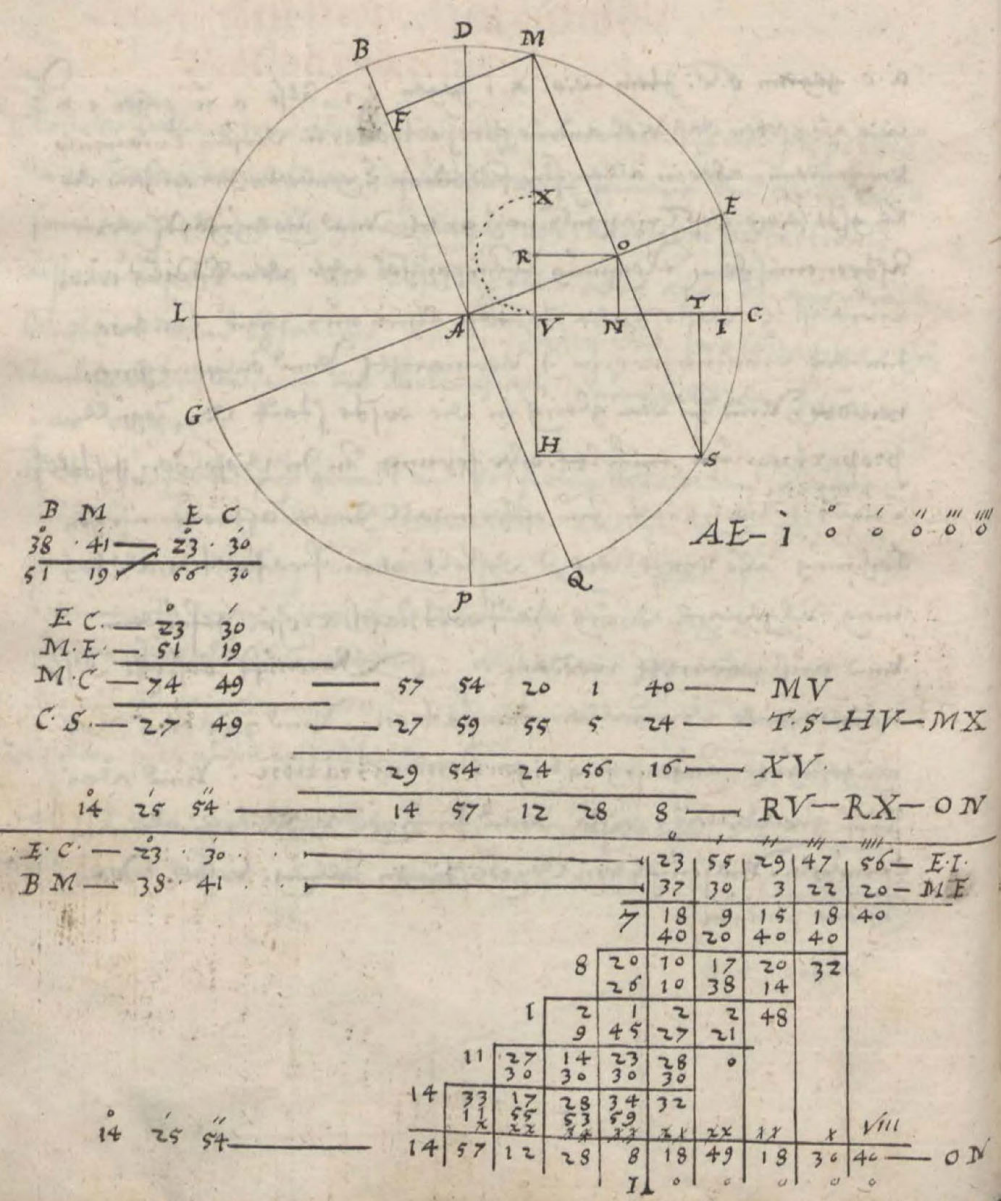
The proof of *prosthaphaeresis* in Bürgi (1592): *Fundamentum Astronomiae*, Wroclaw University Library, Sig. IV Q 38a, fol. 18v

The core of Bürgi’s demonstration of *prosthaphaeresis* consists of a geometrical diagram that is accompanied by a calculation using a set of numerical values, namely 23°30′ for the arc *EC* and 38°41′ for the arc *BM* (Fig. 2, top).^9^ There is no additional explanation or text given in support of it. As can be seen, the specific geometrical representation of the multiplication of two sines given above provides him with the basis for the demonstration. Here, *EI* denotes the sine of *EC*, whereas *MF* is the sine of *BM*. What is not discussed by Launert (2015), however, is that instead of viewing their multiplication as resulting in a rectangular area with sides *EI* and *MF*, Bürgi mounts the lens he had grounded beforehand. *MF* is projected on the radius *AE—*which he as usually sets equal to one—giving rise to its proportional reduction to *AO*, and of *EI* to *ON*. Such a perspective allows him to interpret *ON* as the product of the two sines *EI* and *MF*, corresponding to the arcs *EC* and *BM*, respectively. Hence, in this view, the multiplication by a sine appears simply as an act of proportional reduction.

Launert (2015, 24) goes on to translate the diagram with the calculation into a formula: the arc *ME* is given by subtracting the arc *BM* from a right angle, which, by adding the arc *EC*, gives rise to the arc *MC*, whose sine value *MV* can be determined with the help of a sine table. Analogously, the arc *CS* and thus its sine *TS* can be found. Since *MO* and the angle *OMR* are equal to *OS* and the angle *OST*, respectively, *MR* is equal to the sum of *ON* and *TS*. Hence subtracting *MX* (equal to *TS*) from *MV* allows *ON* to be determined as half the difference between the two. In summary, we arrive at what in modern notation can be expressed as

$$ ON = {\text{sin}}\left( {EC} \right) \times {\text{sin}}\left( {BM} \right) = \frac{1}{2}\left[ {{\text{sin}}\left( {90^\circ + EC - BM} \right) - {\text{sin}}\left( {90^\circ - EC - BM} \right)} \right]. $$

By allowing for the substitution of multiplication by additions and subtractions using a sine table, this relation provides the basis for the method of *prosthaphaeresis*, which can be used to simplify computations. It is in fact just another way of writing what is nowadays known as one of the trigonometric product formulas

$$ {\text{sin}}\left( \alpha \right) \times {\text{sin}}\left( \beta \right) = \frac{1}{2}\left[ {{\text{cos}}\left( {\alpha - \beta } \right) - {\text{cos}}\left( {\alpha + \beta } \right)} \right]. $$

As can be seen from his demonstration, but is a general feature of the treatise, Bürgi restricts himself to the use of the sine only, because this frees the astronomer of the need to have multiple trigonometric tables at hand. As he states it: “the *Canoni foecundo*^10^ are therefore given holidays” (Launert 2015, 109, my emphasis).

This demonstration is referred to as Bürgi’s proof of *prosthaphaeresis* (e.g., Launert 2015, 23; Staudacher 2018, 187). However, rather than simply a geometrical proof of a theorem of pure mathematics formulated for the sake of itself, in the context of early modern astronomy, it must be seen as a response to the computational problem. In other words, it was motivated by and emerged from the mathematical investigation of a technical task an astronomer encountered in his astronomical calculations. On the one hand, the worked example, that is, the usage of a specific set of values, hints at the intention to apply this theorem to numerical, especially astronomical computations. On the other hand, adding the calculation of the product of the sines in the classical non-prosthaphaeretic way below (Fig. 2, bottom) allows Bürgi to illustrate the reduction in computational effort that the replacement of the multiplication of sines by addition and subtraction entails.

By that time, the method of *prosthaphaeresis* was already known to Tycho Brahe, who used it in his own astronomical calculations (Van Brummelen 2009, 265). While it appears to have reached Bürgi through Paul Wittich, a mathematician who stayed in Kassel in 1584 (Staudacher 2018, 147), in this context, the proof presented above, among other things, is assumed to be an original contribution by Bürgi himself (e.g., Launert 2015, 22–23; Thoren 1988). As illustrated above, I argued that the crucial step lies in the specific way of representing the product of sines geometrically. In consequence, let us examine his notion of the sine more closely.

In the next chapter of the *Fundamentum Astronomiae*, he defines the sine in the following manner:

the sine is a straight line from the terminus or end point of an arc in the quadrant running parallel to one leg of the quadrant, but perpendicular to the other leg, and the same legs of the quadrant are radii of the whole circle (Launert 2015, 30).

His statement is clearly aligned with what according to Van Brummelen (2021) was one of the two predominant conceptions of the sine at that time. As far back as the fifteenth century, Regiomontanus referred to the sine as “half-chord […] of the half-arc” (4). The notion of the sine, just as Bürgi’s, was hence tightly tied to circles. Moreover, it is crucial that according to Van Brummelen, Regiomontanus “defines the trigonometric functions as lengths of line segments in the diagrams, not as ratios.” (5).

In Bürgi’s proof of *prosthaphaeresis*, however, this geometrical conception comes up against its limits. As we have already hinted at, if Bürgi were strictly committed to Regiomontanus’ notion of the sine as a line in a circle, he would have represented the product of two sines as an area, rather than a proportionally reduced line. At least for one of the sine factors, Bürgi implicitly goes beyond the confines of this classical definition and adopts a new notion of the sine. Interpreting the sine thus as a proportion allows him to construct the geometrical demonstration in the manner presented above. Let us keep this in mind while we continue our journey through Bürgi’s mathematics, as we will return to it later.

*Prosthaphaeresis* can indeed be seen as offering a solution to the computational problem. [And Bürgi’s proof might have contributed to him being employed by Rudolf II as an imperial clockmaker in Prague in 1604 (Staudacher 2018, 179).] Its actual application, however, hinges on the availability of an adequate sine table. Thus, the reliance on this method creates a subsequent, second-order computational problem, namely, how to determine specific sine values? Or, since this is again subject to a lot of computational effort, how to construct a sine table most efficiently?

It is already in the following chapters that Bürgi commences on his quest to find new tools for precisely this task. Apart from discussing traditional geometric ways of finding sine values, which he finds “just as uncertain and dilapidated, that is, arduous and laborious” (Launert 2015, 42), he outlines a qualitatively different procedure to construct a sine table “by dividing an angle into as many parts as one wants”. This unique method has become known as Bürgi’s *artificium.* The interested reader is referred to the reference given earlier, while we turn to another of his mathematical manuscripts.

## A justification of the occurrence of multiple solutions in algebra

Although not published, Bürgi’s next text was probably written sometime between 1596 and 1603, when it was given to Kepler for revision (Staudacher 2018, 199–201). The manuscript has no title, but is usually referred to as his *Coss* or algebra, as it deals with the application of algebraic tools to the construction of a table of sines. Besides his *artificium*, Bürgi thus manages to identify another method that allows him to divide an angle by any number, that is, finding relations between the lengths of chords of different angles. Instead of being limited to angular bisection, which is the case for classical geometry, algebra proves capable of doing a trisection of an angle and even beyond.

**Fig. 3:**
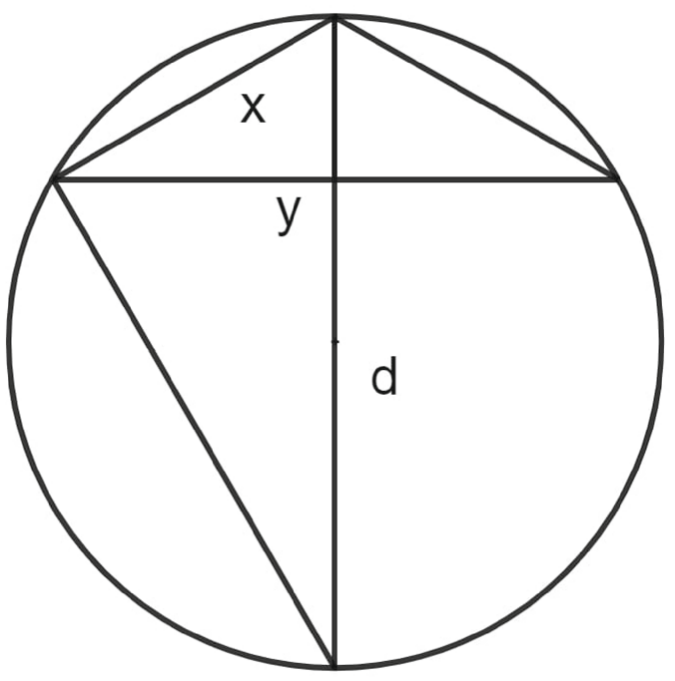
Diagram adapted from Bürgi’s *Coss* as presented in List and Bialas (1973, 28)

Here, I will follow the reconstructions offered by the edition of List and Bialas (1973). After a brief introduction into algebraic entities and the way to deal with these terms (9–22), Bürgi presents the first question with which he is concerned: “If the chord of an arc is known, how to find the chord of half of the arc using the coss” (28)? In other words, what is the algebraic relation between *y* and *x* (Fig. 3)?

Using standard geometrical identities, Bürgi arrives at

$$ y = 2\sqrt {x^{2} - \frac{{x^{4} }}{{d^{2} }}} . $$

He sets the diameter *d* equal to 2, which results in the following algebraic expression for angular bisection (List and Bialas 1973, 28–29):

$$ y = \sqrt {4x^{2} - x^{4} } . $$

In an analogous manner, Bürgi geometrically demonstrates that the chords in the case of the division of the angle by three, four, or five relate just like *x* to $3x - x^{3}$, $\sqrt {16x^{2} - 20x^{4} + 8x^{6} - x^{8} }$ or $5x - 5x^{3} + x^{5}$, respectively. Instead of going on like this indefinitely, however, he creates a table from these expressions (Table 1). By figuring out the regularities of the entries, he simply derives all the following expressions from the previous ones by addition of coefficients and change of signs (List and Bialas 1973, 33–34). Bürgi neither explains why we should expect this kind of regularity, nor does he give a test or provide a proof of the correct workings of this table.

**Table 1:** A part of Jost Bürgi’s table in his *Coss* as reproduced in List and Bialas (1973, 34)

${\varvec{x}}$	${\varvec{x}}^{2}$	${\varvec{x}}^{3}$	${\varvec{x}}^{4}$	${\varvec{x}}^{5}$	${\varvec{x}}^{6}$	${\varvec{x}}^{7}$	${\varvec{x}}^{8}$	${\varvec{x}}^{9}$	**…**
1									
√	1								
3	−	1							
√	4	−	1						
5	−	5	+	1					
√	9	−	6	+	1				
7	−	14	+	7	−	1			
√	16	−	20	+	8	−	1		
9	−	30	+	27	−	9	+	1	
…	…	…	…	…	…	…	…	…	…

The rows are to be read as $x, \sqrt {x^{2} } , 3x - 1x^{3} , \sqrt {4x^{2} - x^{4} }$, etc. While the third, fourth, fifth, seventh, eighth, and ninth rows refer to angular trisection, bisection, division by five, seven, four, and nine, respectively, the entries in the second and sixth rows do not have any meaning in these terms

Using these algebraic expressions, it is possible to compute a precise value for the sine of one degree, which was undoable purely via geometrical means. For instance, by dividing the chord of 60°, which is equal to one if the diameter is set to two, continually by five, three, and two, one can calculate the chord of two degrees. Despite these advantages, there have been at least two kinds of objections toward the use of algebra in this context. To illustrate them, let us consider the quintisection of the chord of 60°, which Bürgi discusses himself (List and Bialas 1973, 37). The equation to be solved for *x*, that is, the chord of 12°, reads

$$ {\text{crd}}\left( {60^\circ } \right) = 1 = 5x - 5x^{3} + x^{5} . $$

First, by looking at the first two terms of the expression, “a geometer might object to the cossist and say” (List and Bialas 1973, 30):

How can a length or a line be diminished by a cube or a bodily quantity^11^? Is this not just as if I said three cubits less one *seidl*^12^? (30, *my emphasis*).

Bürgi’s response to this issue is as straightforward as it is simple.^13^ Either of them must be understood arithmetically as being constructed from units. In his words, since “thus both the length and the cube [are] each a number, which is why number can very well be compared with number” (30).

Second, we would expect a polynomial of degree five to possess five solutions. But how can the angular division of a chord lead to multiple solutions? Sine values must be uniquely defined. This is one of the points of critique that Johannes Kepler (1619) uttered about Bürgi’s algebra in his *Harmonice Mundi* (Bos 2001, 191; Launert 2011; Ullrich 2021). To salvage the algebraic treatment of chords, Bürgi offers a conceptual interpretation of this issue (List and Bialas 1973, 36–38). Despite having argued before that one is here dealing solely with arithmetical numbers, he reintroduces the geometrical notions of the sine and chord as lengths in a circle to justify the occurrence of multiple solutions in algebra.

Once we set the algebraic expression $5x - 5x^{3} + x^{5}$ equal to one, Bürgi reminds us, we in fact merely specified the length of the initial chord to be quintisected, not, however, the angle it corresponds to. Since a chord of length one is not only associated with an arc of 60°, but just as well with one of 300°, quintisection should result in the chords of both 12° and 60°, respectively. Moreover, we can “add one or two full circles” (List and Bialas 1973, 37) to these two arcs and subsequently divide them by five

$$\begin{aligned}& \frac{60^\circ + 360^\circ }{5} = 84^\circ ;\frac{300^\circ + 360^\circ }{5} = 132^\circ ;\\& \frac{60^\circ + 2*360^\circ }{5} = 156^\circ ;\frac{300^\circ + 2*360^\circ }{5} = 204^\circ . \end{aligned}$$

As the last two angles again refer to a chord of the same length—as would all the following expressions—we arrive at exactly five outcomes.^14^

In the introduction to his *Coss*, Bürgi explicitly mentions Van Ceulen’s (1596) book *Vanden Circkel* as a source of inspiration. Although it is not fully clear how its content reached him,^15^ it does contain derivations of algebraic expressions for all kinds of angular divisions. Unlike Bürgi, however, Van Ceulen did not seem to realize the peculiar feature of their coefficients that makes it possible to reach higher expressions solely by means of simple operations in a table. Furthermore, the justification for the occurrence of multiple solutions is also absent. It is thus plausible to assume that this interpretative idea was an original contribution by Bürgi himself. Once again, let us keep it in mind to save it for later and go on with his mathematical adventures.

In the introduction to his *Coss*, Bürgi also states that he intends to use algebra together with his *artificium* to construct an enormous sine table with values for every even second (List and Bialas 1973, 8), which would have been the most detailed and accurate of its time (see Van Brummelen 2021, 17). Although his foster son Benjamin Bramer (1648) tells us that Bürgi did calculate the table, there is no direct evidence (see List and Bialas 1973, 112–13, which also includes some more indirect evidence). While the effort needed for the computation of values for every two seconds would in any case have been huge, Bürgi’s new tools had the capacity to reduce it a lot. In comparison, even though its resolution was five times lower, the construction of the *Opus Palatinum de triangulis* by Georg Joachim Rheticus and Valentinus Otho (1596), which became the most extensive sine table in usage, took Rheticus more than his lifetime, although he partially employed a team of computers (see Burmeister 1968, III:187–90)—it was only finished after his death by his pupil Otho—because it was based solely on geometrical methods (Van Brummelen 2009, 273–83).

Concerning the potential use of such a table, Lutstorf and Walter (1992, 9–10), among others, have already offered an explanation. They asserted that the original idea behind it was not so much its application in astronomical calculation—for which it was too detailed and accurate, but to use it in combination with the method of *prosthaphaeresis* as a general means to multiply numbers. By adjusting its decimal dimension (e.g., 12,345 as 0.12345), it is possible to treat any number as the sine value of a specific angle. This in turn would allow the application of *prosthaphaeresis* as a tool to simplify any multiplication of two many-digit numbers, such as might have shown up in fields such as surveying, gunnery, or navigation, which became subject to trigonometrization by the end of the sixteenth century (Van Brummelen 2021, 45–61). The larger the sine table at hand is, or in effect, the smaller the step size and the more positions given to the entries, the longer could be the numbers that can be multiplied without having to go through an inefficient interpolation procedure.

I located three additional hints in Bürgi’s mathematical oeuvre that support this explanation. First, when Bürgi introduces the method of *prosthaphaeresis* in the *Fundamentum Astronomiae* in the passage quoted above, instead of motivating it via the problem of sine value multiplication, he connects it to a proportion of numbers in general. Second, in his *Coss*, Bürgi mentions that, according to Wittich, “the general sine tables were not sharp enough” or contained too many mistakes (List and Bialas 1973, 7), which is why he intends to construct a more accurate one. However, he makes this statement not in relation to astronomy, but with respect to the method of *prosthaphaeresis*.^16^ Third, Bürgi’s last mathematical invention—to which we will now turn—can be characterized as a solution to the problem of finding a general means for simplifying calculations. We can thus assume that this task was on his horizon from the onset.

## A logarithmic computational tool and the presentation of “the whole red number”

In the light of having to perform operations such as multiplication, division, or root extraction, Bürgi mentions that “the multitude of tables” one needs to have at hand “is not only annoying but also cumbersome and difficult” (Clark 2015, 121). From thence arises his motivation “to invent general tables with which you could do all of the above-mentioned things” (58^17^). This is what Bürgi tells us in the handwritten foreword to his *Arithmetische und Geometrische Progress Tabulen* that were printed in 1620.^18^ I will follow the reconstruction in the edition by Clark (2015) to briefly describe the overall working of this piece of mathematics to then focus on one specific aspect of it.

**Fig. 4:**
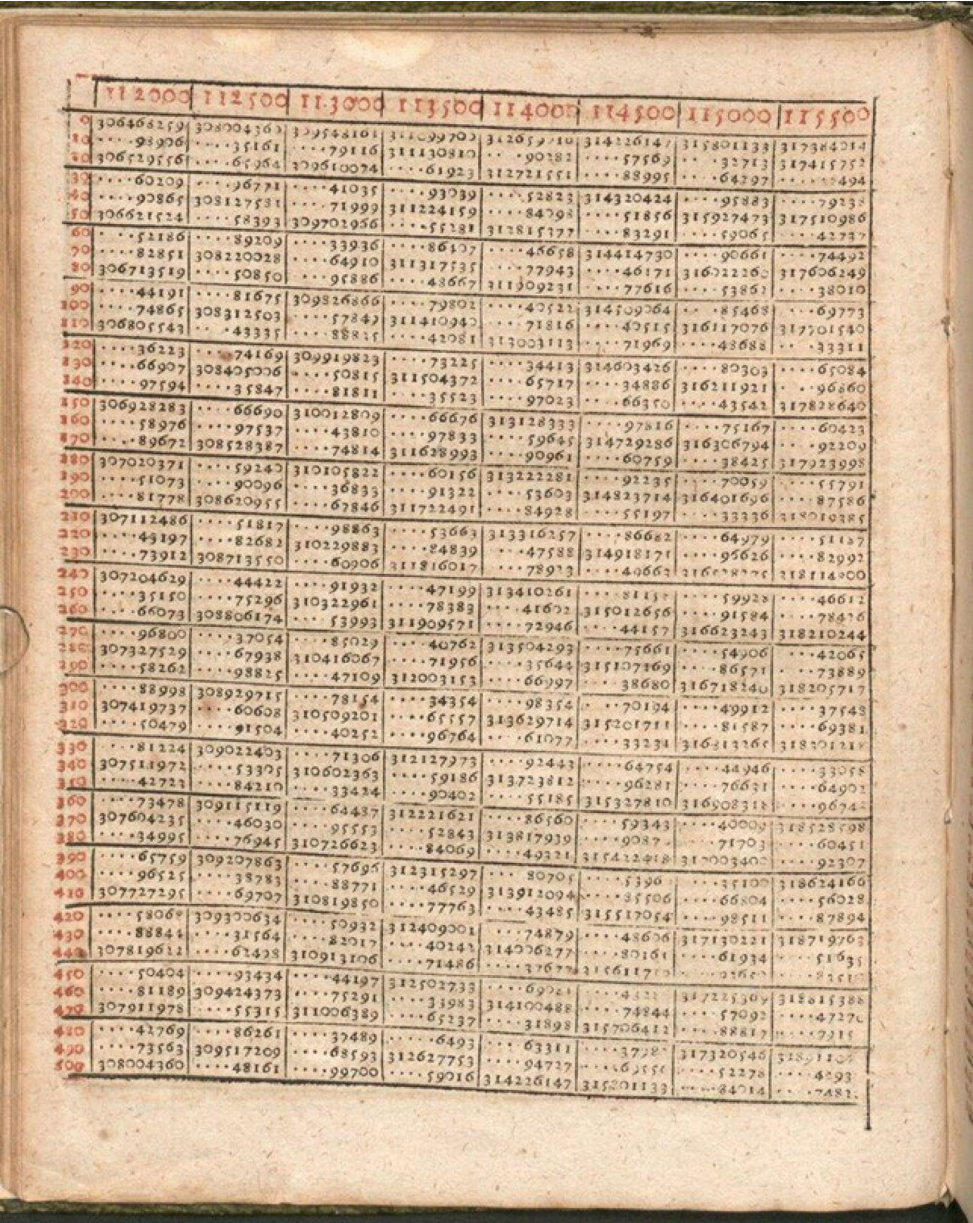
One page of Jost Bürgi’s (1620): *Arithmetische und Geometrische Progress Tabulen*, Bavarian State Library, Res/4 Math.p. 55 w, 30

The tables constitute a logarithmic computational tool. Over 58 pages, they list the powers of the base 1.0001 (multiplied by 10^8^), that is, a geometrical sequence as black numbers. The corresponding arithmetically progressing red numbers—the logarithms so to speak—are given as the argument (Fig. 4). Some extant versions of the tables include short instructions, in which Bürgi goes through explicit numerical examples to illustrate how various kinds of calculations can be simplified with it.

The procedure is always similar: First, the numbers of the initial calculation are located as black numbers in the tables and their associated red numbers are identified.^19^ Due to their logarithmic properties, multiplications or divisions thereby result in simple additions or subtractions, while root extraction or squaring becomes halving or doubling, respectively. Hence, second, the simplified operation is performed, and third, the resulting red number is transformed back again into a black number.

**Fig. 5:**
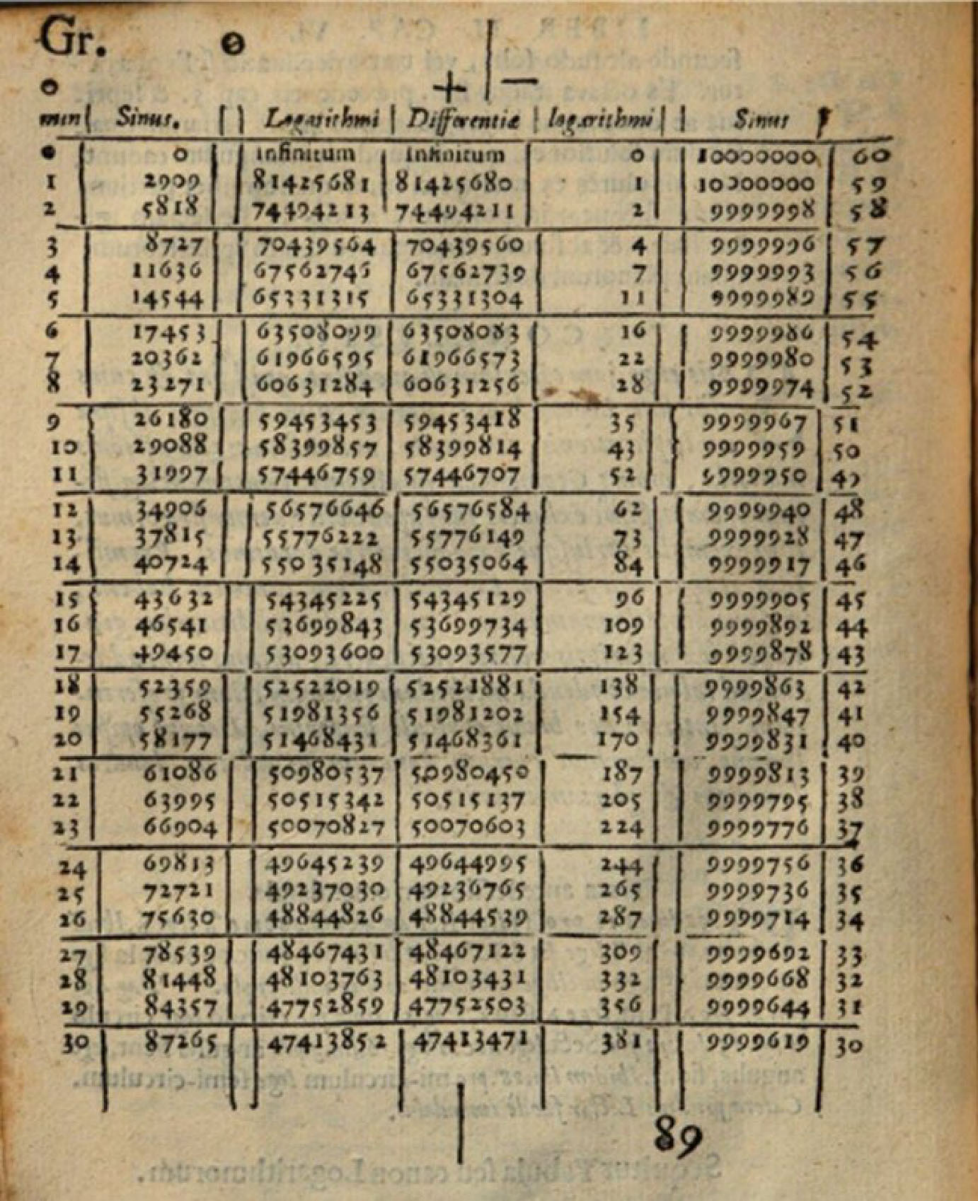
First page of John Napier’s (1614): *Mirificum Logarithmorum Canonis Descriptio*, Regional State Library of Regensburg, 999/Philos.1791, 58

Although printed 6 years after the publication of John Napier’s (1614) *Mirificum Logarithmorum Canonis Descriptio*, which introduced logarithms into the world, scholars generally agree that Bürgi developed his tables independently of him (e.g., Clark and Montelle 2012). There is also a major difference between Napier’s and Bürgi’s versions. While Napier tabulated the logarithms of sine values (Fig. 5), exemplifying their intended application in spherical astronomy or trigonometry more generally (Van Brummelen 2021, 62–68), Bürgi lists bare numbers.^20^ This, however, implies an extra difficulty.

The black numbers in Bürgi’s tables range from 1 to 10; hence, they are not sensitive toward the decimal dimension. In other words, the black numbers of 0.5, 5, and 50 are all associated with the same red number. While the tool can thus present the correct string of digits of the result, the decimal point must be set manually. However, there can be a further complication connected to it, as shows up in the following instructive calculation of Bürgi:

Find the mean proportional between 2 given numbers. It is, however, that the 2 given numbers do not have an equal number of digits. So the first has 7 digits and the other has 8; the first [one] is 2447471 and the other 33033604 (Clark 2015, 157, additions in the original).

As explained by Clark (2015, 163–66), in this case, one cannot simply half the sum of their corresponding red numbers—as one could do if they had the same dimension. One would end up with the mean proportional between 2′447′471 and 3′303′360,4 or 24′474′710 and 33′033′604, respectively, depending on how the result is interpreted. To deal with this incongruence instead, Bürgi tells us that “the whole red number” (17), that is, 230′270,022, which corresponds to the black number of ten in Bürgi’s basis, must be added to the red number of the larger number to account for the different number of digits. Analogously, if the red number after a computation exceeds the whole red number, it must be subtracted continuously, until the red number drops below this threshold, thereby always adding a factor of ten to the result.

**Fig. 6:**
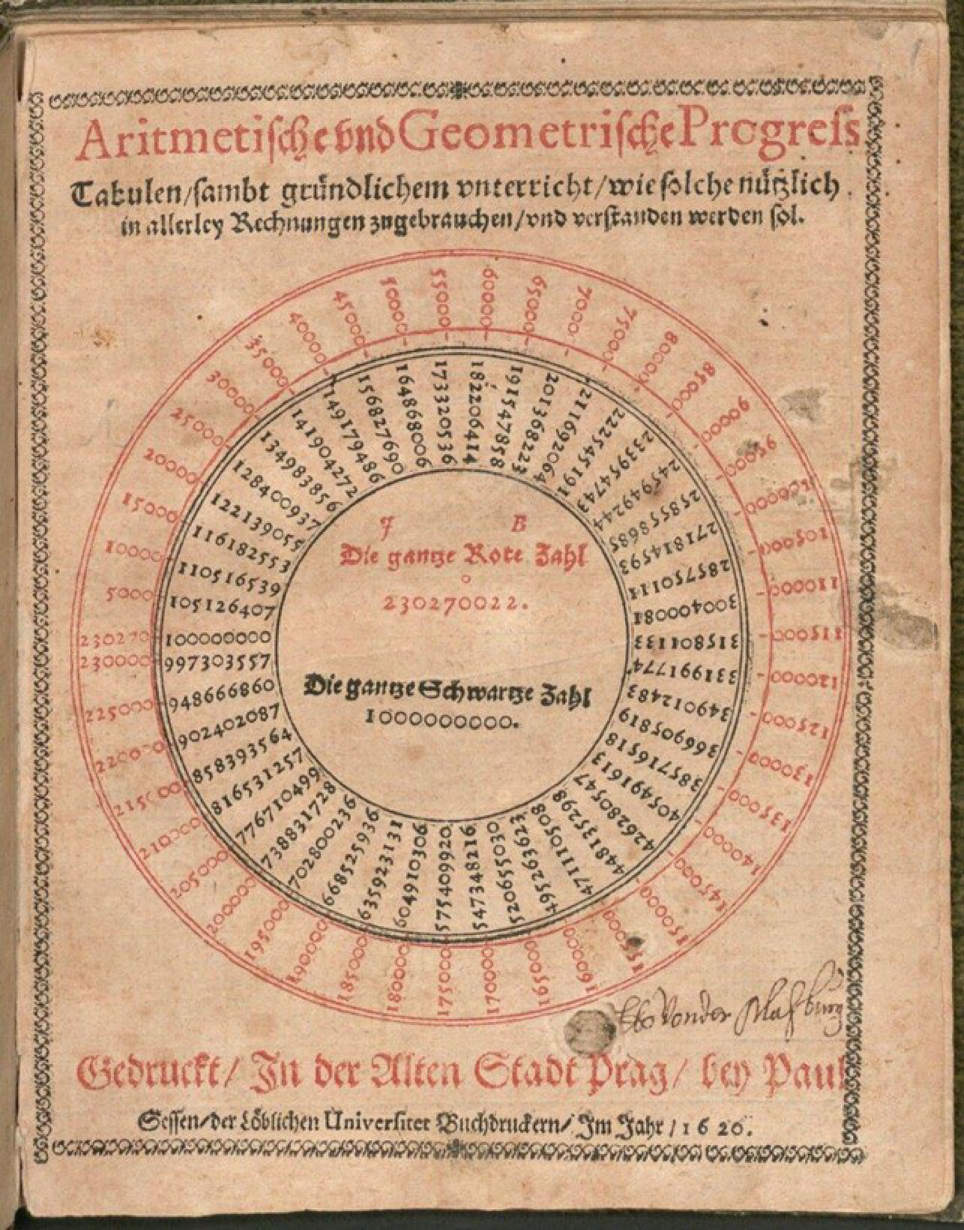
Title page of Bürgi’s (1620): *Arithmetische und Geometrische Progress Tabulen*, Bavarian State Library, Res/4 Math.p. 55 w, 1

**Fig. 7:**
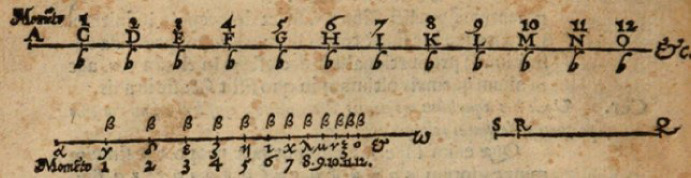
Illustration of the logarithmic properties in Napier’s (1614): *Mirificum Logarithmorum Canonis Descriptio*, Regional State Library of Regensburg, 999/Philos.1791, 4

Bürgi probably realized that the issues with the whole red number could prove to be the conceptually most difficult features of his tables for users to grasp, potentially complicating their applicability. I assume that this is the reason why the title page of his printed mathematical work is dedicated to its representation (Fig. 6). As mentioned by Waldvogel (2014), the circular depiction visualizes the effects of the decimal shift. This stands in stark contrast to the way Napier presented his logarithms. He could completely circumvent the problem of decimal dimensions by directly listing sine values. Thus, they are presented with reference to different rates of increase of one-dimensional geometrical magnitudes (Fig. 7), as described by Van Brummelen (2021, 64–65), rather than in a circular manner.

In 1631, Bürgi moved back from Prague to Kassel and died in the following year at the respectable age of 79 (Staudacher 2018, 294). Among others, he left posterity with the three mathematical texts presented above.

## A clockmaker’s mathematics

Bürgi’s mathematics, according to the way I presented it here, confirms the point of view of the Hessen–Grossmann Thesis that emphasizes the interaction between science and technology. His case illustrates a close relation between the mathematical and technological developments at that time. The advancements in fine metal working around 1600, during what has been described as “the golden age of German clockmaking” (Maurice and Mayr 1980), which is exemplified by Bürgi’s increasingly precise clocks and measuring instruments, and the application of these tools to astronomical observation in early modern Europe, for instance, laid the foundation for the occurrence of the computational problem. The growing interest in the dynamics of the heavens thus entailed not only an improvement of mechanical, but also a scrutiny of the mathematical tools necessary for this endeavor. Along this line, we can comprehend Bürgi’s proof of *prosthaphaeresis* and, connected to it, his quest of finding new ways to construct a sine table, which included his *artificium* and his elaborations on the tools of algebra. With his logarithmic computational tool, he even invented a general means to simplify computation of various sorts, promising applications not only in astronomy but all mathematized fields.

It is necessary to stress, however, that although possible in principle, apparently not even the handiest computational tool of that time—the logarithms—found any substantial usage in fields, such as surveying, gunnery, or navigation.^21^ The reason for this was simply the missing ability and demand to reproduce results at such a high level of precision that would have made the application of these tables profitable (Schneider 1970).^22^ In accordance with the Hessen–Grossmann Thesis, I thus argue that, while emerging from the study of problems faced in technical practices, Bürgi’s mathematics retained a relative autonomy with respect to these concerns. His works formed their own discourse within the structure of practical knowledge that is not reducible to technology. Constructing an immense sine table in less time, for instance, could serve as the proof of the advantages of the mathematical methods he developed and employed, rather than actually reducing the burden of multiplying numbers in a practitioner’s life.

The perspective on mathematics as a contingent human practice furthermore posits that the newly emerging questions and material to be studied mathematically do not by themselves fully determine the specific way they are addressed. The current state of mathematical knowledge does not strictly define its future application into novel fields of inquiry. For instance, even though, as we have seen earlier, Bürgi shared the common conception of the sine as a line in a circle—at least in words—when confronted with the task of giving an explicit geometrical representation of the multiplication of two sines to demonstrate the method of *prosthaphaeresis*,^23^ he opts for a proportionally reduced line instead of a rectangular area in a circle that would be more true to meaning of the definition. In the framework of the Hessen–Grossman Thesis, we thus face the open question of how to conclusively explain the occurrence of these kinds of conceptual shifts.

While Omodeo (2019) suggests integrating the ideological sphere into the Hessen–Grossmann Thesis, I do not want to focus on the socio-political dimension here.^24^ Instead, I wish to propose a new technology-based approach to analyze and account for the developments in mathematics that can be traced back to the work of technician–mathematicians. It is based on the assumption that their practical engagement with technology shaped their mathematical concepts and practices. In the case of Bürgi, I argue that some of his original contributions to mathematics reveal a connection to his technical practice as a clockmaker, especially his interaction with clockwork mechanism and the tools used to construct them.

Let me introduce my method and corroborate my claim by applying it to the three aspects of Bürgi’s mathematics, around which the presentation of his mathematical works given above was grouped. These include his new notion of the sine inherent to his proof of *prosthaphaeresis*, his way of geometrically justifying multiple solutions in algebra and his circular depiction of the whole red number in his tables of progressions. The reason for picking them is, among other things, that he covered new ground in these instances, that is, he could not just follow preestablished mathematical procedures to arrive at them. Consequently, there was a certain degree of contingency inherent to these steps, which is why we can expect them to bear his own signature, that is, the signature of a technician.

The first and most apparent hint that Bürgi’s technical profession—especially clockmaking—shaped the way he looked at mathematical entities can be found on the title page to his logarithmic computational tool (Fig. 6). The circular representation of the whole red number not only resembles the dial of a clock. It even works in a similar way. If the second hand^25^ makes a full turn, the minute hand takes one leap. Analogously, if the red number goes beyond 230′270,022, that is, if it completes a full circle, it drops to zero again and the decimal point of the result shifts one position to the right. Such a point of view suggests that as Bürgi faced the task of conceptually illustrating the effect of the whole red number when constructing his tables of progressions, he was guided by his technical practice.

The potential origin of Bürgi’s way of interpreting the occurrence of multiple solutions in algebra is again connected to clockmaking, specifically to his practical experience of dealing with cogwheel mechanisms. Stumbling upon the fact that finding the chord of *α* by angular division of the chord of *nα* via algebraic expressions results in up to *n* different solutions (for *n* ≥ 1), he argues that the chord of the angle *nα* is associated with several different angles, which are all of the form $m*360^\circ \pm n\alpha$ (for *m* ≥ 0). The interpretation that the given length of a chord does not strictly define the angle it corresponds to is structurally analogous to a mechanism, in which an initial cogwheel is repeatedly given a full turn, making another cogwheel end up in a variety of distinct positions. In this case, the final state of the initial cogwheel does not uniquely determine the outcome. There are several different possibilities that open up, just as is the case with the original chord. Hence, I argue that Bürgi’s justification might derive from an experience he constantly made as a clockmaker.

**Fig. 8:**
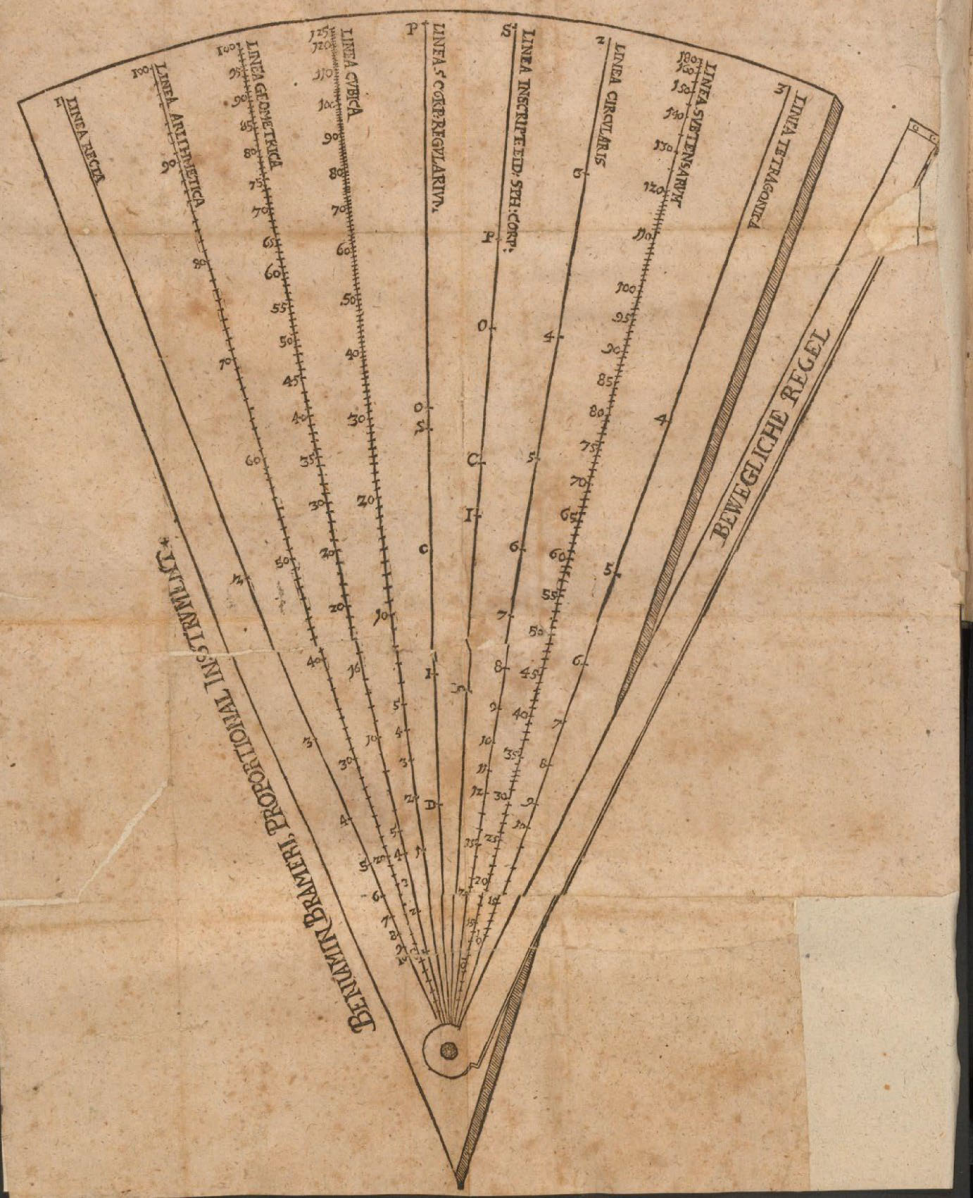
Illustration of the proportional instrument presented in Benjamin Bramer’s (1615): *Beschreibunge vnd Underricht/Wie allerley Theylungen zu den Mathematischen Instrumenten zu verfertigen: Neben dem Gebrauch eines newen Proportional Instruments,* Zentralbibliothek Zürich, NE 2229,2, fol. 55

**Fig. 9:**
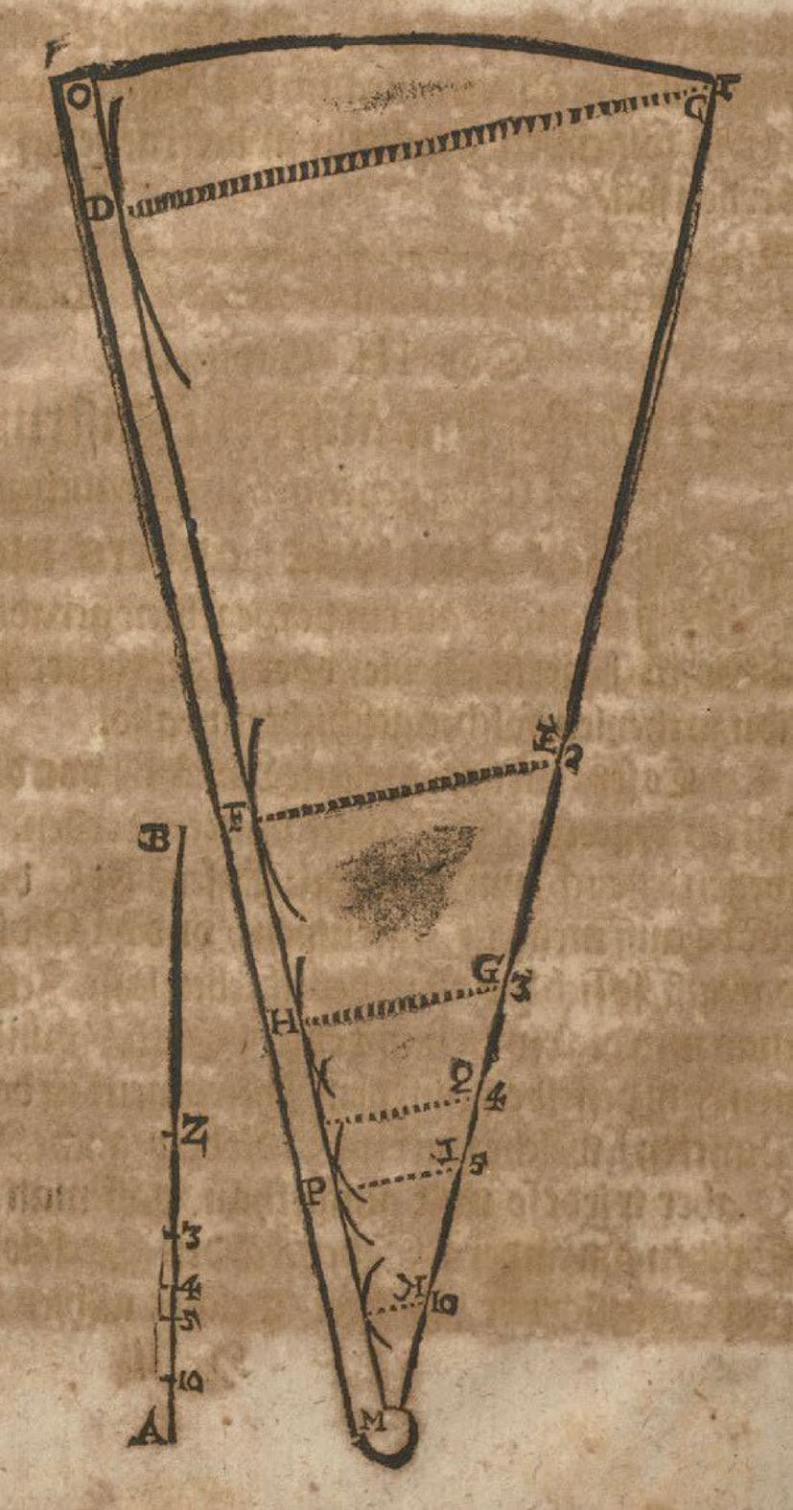
Illustration of the working of the proportional instrument in Benjamin Bramer (1615): *Beschreibunge vnd Underricht/ Wie allerley Theylungen zu den Mathematischen Instrumenten zu verfertigen: Neben dem Gebrauch eines newen Proportional Instruments …*, Zentralbibliothek Zürich, NE 2229,2, fol. 60

A similar interaction can be identified for his geometric representation of the multiplication of sines. Making cogwheels with a given number of teeth is a crucial part of a clockmaker’s job. To determine its size, one could use a proportional instrument, as the diameter must be proportional to the number of teeth if their modus is kept fixed. Bramer’s proportional plates can be seen as an illustrative example of such an instrument. It consists of a movable ruler on plate on which lines with specific measures are inscribed (Fig. 8). Although this specific instrument was published only in 1615, Bürgi must have worked with similar kinds already earlier as he himself invented a new version of a proportional instrument, the proportional circle much before that.^26^ The workings of these instruments are all based on some form of the intercept theorem. In Bramer’s case, for instance, the movable ruler is opened so much that it can just be reached by the original length, taken with a compass and measured from the corresponding marking on (one of) the line(s)—from the point *C* at the marking of 1 in this case (Fig. 9). If one now wants to take half this length, one can simply take the distance between the ruler and point *E* at 2 with a compass. Depending on the division of the line, the instrument allows for various applications—determining the size of cogwheels from the proportion of their number of teeth, for instance, can be done using the linear line *linea arithmetica*.

The crucial point to be made here becomes apparent if Figs. 1 and 9 are compared. The way Bürgi geometrically represents the multiplication of sines in his proof of prosthaphaeresis is identical to the way the instrument works. If a length (or a sine) is multiplied by a sine value, that is, the line to be multiplied is taken as the distance between the marking of the full radius and the movable ruler—Bramer’s proportional plates in fact also include a line divided according to the length of the relative chords up to 180°, the *linea subtensarum*—then the result is given as the distance between the movable ruler and the respective sine value. In other words, the instrument yields a proportionally diminished line as the result of its multiplication with a sine value—in line with Bürgi’s understanding and reference of multiplication as a “proportional operation”—which can be extracted with a compass. Hence, I suggest the proportional instruments used by technical practitioners at that time to form the material embodiment of his notion of sine multiplication.^27^

In summary, according to my analysis, Bürgi’s practical engagement with technology might have shaped the structure of his geometrical proof of *prosthaphaeresis*, his justification of the occurrence of multiple solutions in algebra, and the aesthetic presentation of a procedural step in his logarithmic computational tool. These three potential instantiations of technical experience from clockmaking in his mathematical works proposed here thus illustrate the hypothesis of this article, namely that Bürgi’s practice as a mathematician portrays a clockmaker’s mathematics.

## Solving polynomials like a “superior craftsman”

To the sceptical reader, I can offer another of Bürgi’s mathematical peculiarities to strengthen my argument. Up to now, one crucial topic of his algebra has been averted, namely how does he solve the expressions to find the length of the chords? They are usually polynomials of a higher degree without any analytic way to determine *x*. Moreover, Bürgi mentions in the introduction to his *Coss* that Van Ceulen “concealed the best and most essential thing in the algebra [, …] namely the equations” (List and Bialas 1973, 7). Hence, we can infer that his approach to finding numerical solutions to algebraic equations expresses once again his own handwriting, that is, his particular way of approaching mathematical problems.

Bürgi starts with a general rule for how to solve an algebraic equation for *x*: Assume that you know *x*, insert it into the expression and check if it is correct. If not, you have at least figured out what *x* would have been in case the chord was of the value you now reached. This procedure allows you to approximate the solution by finding continually closer upper and lower bounds to the result (List and Bialas 1973, 43–48). To enhance the guesswork and thus speed up the process, Bürgi proposes to find a first approximation for the length of the chord by means of a mechanical device (49–50). Specifically, the chord of the divided angle is drawn, and its length in comparison to the radius is simply measured. While some mathematicians at that time opposed the introduction of approximative methods within mathematics, Bürgi went a step in the opposite direction. Just like an artisan, he advises us to rely on a mechanical tool in our mathematical task.

From a modern perspective, another feature, reconstructed by List and Bialas (1973, 68–69), appears even more striking. Confronted with the algebraic expression of angular bisection, namely $\sqrt {4x^{2} - x^{4} }$, it is obvious to check if there is a way to get rid of the root. However, according to the rules governing root extraction of algebraic expressions, which Bürgi introduced earlier in his *Coss* (16–19), it can only be done if the expression possesses an odd number of terms. He therefore suggests adding 0 as a bare number term and reordering the terms to $\sqrt { - x^{4} + 4x^{2} + 0}$.

To him, however, there is no term that results in $- x^{4}$ if squared. To resolve this issue, he states: “I realized that I should not look at the signs. [I] therefore let go of them” (List and Bialas 1973, 69). But still, the root of $\sqrt {x^{4} + 4x^{2} + 0}$ cannot be gotten rid of. Instead, he figures out that $x^{2} + 2$ would be the root of $x^{4} + 4x^{2} + 4$, and hence we can—by letting go of the signs—assume the following relation:

$$ \sqrt { - x^{4} + 4x^{2} + 4} = x^{2} + 2. $$

If the initial chord to be bisected is equal to zero, Bürgi tells us, our starting point is the algebraic equation

$$ \sqrt { - x^{4} + 4x^{2} } = 0, $$

and thus

$$ - x^{4} + 4x^{2} = 0. $$

One could easily solve it for *x*, which is not, however, what Bürgi has in mind. Rather, he tells us to add 4 on both sides and again take the root

$$ \sqrt { - x^{4} + 4x^{2} + 4} = \sqrt 4 = 2. $$

Since we know, according to his previous elaborations, how to resolve the left-hand side of the equation, we end up with the following expression:

$$ x^{2} + 2 = 2. $$

Bürgi subtracts 2 on both sides and explains that this reveals $x^{2}$ to be equal to “the squared chord of 0” (List and Bialas 1973, 69). It can be concluded, according to him, that the correct algebraic expression of the chord *x* must be $\sqrt {x^{2} }$.

One might be tempted to ask what the use of this derivation (which from a modern point of view contains at least three major mathematical faux pas) should be? It obviously cannot provide us with any insightful result, apart from the trivial fact that the cord of the angle zero remains invariant upon bisection. Bürgi’s intention behind presenting this argument in his *Coss* is accordingly not a utilitarian one. Instead, he tells us to return to his table of algebraic expressions (Table 1), to realize that $\sqrt {x^{2} }$ had been standing there, in the second row all along.

“Lo and behold”, Bürgi states upon this finding, “the same divisions are each in their rightful place […] in this fantastical table, and so this table does more than it has learned from the algebraic processes” (List and Bialas 1973, 69). In other words, the derivation ascribes meaning to an entry in the table, which did not have any interpretation beforehand. Even though this procedure is not applicable, Bürgi thus attempts to settle any remaining doubt about the validity of the table and instead strengthens and justifies it by making it appear even more ingenious.

Coming back to our initial question, what does Bürgi’s treatment of polynomials tell us about his way of doing mathematics? On the one hand, in his mathematical practice, he does not stop or give up when confronted with what in the traditional perspective would be considered a dead end. If an algebraic equation cannot be solved analytically, for instance, instead of dismissing it as bad or imperfect mathematics, he simply approximates the results. On the other hand, he appears to be very undogmatic when it comes to the means to be employed—if it is more efficient, just measure the chord on a sketch. The moment he stumbles upon the root of negative entities, rather than recoiling in fear, he just tries to work his way around the contradicting parts. Thereby, with some trial and error, by experimenting and even stepping over the borders of what is considered mathematically sound multiple times, he manages to provide some meaning to one of the table’s entries. But where does this kind of approach to mathematics come from?

In the 1940s, Edgar Zilsel (1942/2003) proposed his thesis, according to which the transformations in early modern science can be seen as the result of the merger of the methods of skilled artisans with that of educated scholars. In his words, Bürgi can be characterized as a “superior craftsman” (4). Not only does his technical practice rely on mechanical devices and approximations. It can also be characterized by an urge to improve and innovate—it was all about increasing the accuracy and stability of his observational and planetary clocks, celestial globes, and measuring instruments. In consequence, he must have been very appreciative toward new developments and ready to experiment with various techniques to integrate them into his toolbox as a technical practitioner.

I argue that the same goes for his approach to mathematical tasks. Instead of having internalized a long and stable tradition that allegedly went back to ancient Greece, like academically trained scholars such as Kepler or Rheticus, it is not completely clear where Bürgi learned his mathematics. He probably took it up in a more eclectic manner along the way, through the interaction with peers and scholars or even as an autodidact. In consequence, instead of being restricted to conforming with classical standards, he embraced new mathematical tools that might prove to be useful, even if one had to stretch the given conceptual apparatuses. As we have seen, for instance, his mathematics expresses a flexible treatment of the notion of sine. Depending on the situation, it was either half a chord in a circle, a proportion, just a number or even a length on a mechanical device.

Considering the experiential disposition with which Bürgi approached mathematical problems that stems from his technical practice corroborates my hypothesis about his texts portraying a clockmaker’s mathematics. When stepping into unexplored mathematical terrain, there might not yet be any fixed rules on how to deal with the monstrous creatures that reside there. Certain procedural steps are underdetermined from the perspective of established mathematics. Hence, in such a world—if one does not want to leave it for good—one must rely not only on one’s instincts, but also one’s intuition trained from coping with analogous settings, albeit in different contexts. Imagine Bürgi in such a situation, when he conceptualizes the multiplication of two sines, geometrically interprets the occurrence of multiple solutions in algebra or represents the shift in decimal places in logarithmic numbers. Since there was no authority to turn to in these matters at that point in time, it makes perfect sense to expect him to have drawn, among other things, from the knowledge inherent to the technical practitioner’s experience.

## Outlook

In conclusion, the presentation and analysis of Bürgi’s three treatises in mathematics performed in this article suggest that they were strongly affected by technological developments of the time. According to the narrative I presented, the increased accuracy that was reached within early modern astronomy with the help of advanced measuring instruments amplified the computational problem, which in turn was the main factor that motivated and directed his mathematical inquiries. Beyond that, however, I argue that his practical engagement with technology as a clockmaker shaped his mathematics from within, that is, its entities, justifications, presentations, proofs, and procedures. Hence, I called it a clockmaker’s mathematics.

The proposed interaction between the art of clockmaking and mathematical practice that can be encountered in the figure of Bürgi offers not only a potential explanation for the origin of some of his mathematical innovations, but also hints at an additional narrative to account for the transformations in the views and study of nature in early modern Europe. Instead of assuming that given mathematical tools were used for the study of mechanisms and instruments, the mechanical devices themselves might have shaped mathematics via the hands and brains of technician–mathematicians. The integration of knowledge and experience of technical practitioners into mathematical discourse might in turn have facilitated its application to problems of theoretical mechanics, among other things, by taking over some of the necessary translation work, such as slight conceptual shifts and changes of meaning.

The potential emergence of a distinct mathematical and computational culture out of technological craftwork—the upcoming of a distinct genre of a technician’s mathematics in the sixteenth/seventeenth century—invites for further study. The research could strengthen a technology-based perspective on mathematics, which might prove more adequate for our current context, which is, among other things, characterized by the increasing reliance on computational technologies, both in mathematical practice as well as in our everyday lives. My approach to analyze the interrelations between mathematics and technology, which was illustrated around a case study from early modern Europe, but might prove applicable to other contexts as well, can provide an inspiration and methodological tool to contribute to such an endeavor.

## Data Availability

Data sharing is not applicable to this article as no datasets were generated or analyzed during the current study.

## Footnotes

1 The analytic category of the technician-mathematician I introduce is distinct from other figures, such as the *ingénieur savant* defined by Grattan-Guinness (1993), as it is indifferent to the specific mathematical interests of these people.

2 Both have been frequently discussed in secondary literature. For his logarithmic computational tool, which consists of a table that correlates a geometric with an arithmetic progression, see, among others (Clark 2015; Lutstorf 2005; Lutstorf and Walter 1992; Roegel 2010; Waldvogel 2014). The *artificium*, an algorithm for determining sine values by continuous tabular additions, is presented in Folkerts et al. (2016), Launert (2015), Waldvogel (2016). For a discussion of how Bürgi might have come up with it, see Roegel (2015, 2016), Ullrich (2016), Wagner and Hunziker (2019). The latter includes an English translation of the relevant chapter of the manuscript.

3 See Gaulke (2010) for the context that encouraged this kind of endeavor.

4 Wrocław University Library, IV Qu 38a.

5 All translations of Bürgi from the editions by Launert (2015) and List and Bialas (1973) are my own.

6 In fact, at this point, Bürgi did not yet have a way to simplify division (Launert 2015, 29).

7 In the manuscript, it reads *ai*, which is corrected by Launert (2015, 22).

8 In the manuscript, it reads *or*, which is corrected by Launert (2015, 22).

9 The connection of *prosthaphaeresis* to the computational problem of early modern astronomy is further exemplified by the fact that the geometrical demonstration imitates an astronomical diagram. The diameters GE and LC are the celestial equator and ecliptic, respectively, with the standard angle of 23°30’ between them, B and Q are the poles, and M signifies the location of Kassel, which has a latitude of 51°19’.

10 This refers to the “*canon fecundus*” in Erasmus Reinhold’s (1554) *Tabularum directionum* (Launert 2015, 110), which are trigonometric tables portraying multiple trigonometric functions including the tangent (Van Brummelen 2021, 6).

11 The term Bürgi uses is “Cörperliche quantitet”, meaning measure of volume.

12 This is an Austrian unit of capacity used for beer and equivalent to 0.3 L.

13 Although it is not clear where he gets it from.

14 Bürgi does not mention or discuss the meaning of the fact that two of the solutions of this polynomial equation are negative.

15 He mysteriously states that the book “appeared in the German *or* Dutch language” (List and Bialas 1973, 7, my emphasis), but there seems to be no record of a German version and we are not aware of Bürgi having any mastery of Dutch. One assumption I suggest is that he was taught these algebraic methods by his friend Nicolaus Reimers Ursus, who stayed in Kassel in 1586/7 (Launert 1999). By that time, Van Ceulen mentioned that he already knew his algebraic methods for computing π (Katscher 1979, 112). Furthermore, Van Ceulen responds in his book to a cyclometric problem posed by Reimers in 1583, whose later works are also concerned with algebra, although they do not deal with the division of angles and computation of chords (Launert 2007).

16 In 1590, the latest, Bürgi already was in possession of a sine table that was more than accurate enough for all astronomical purposes, as I will show in another forthcoming article.

17 Clark translates “General Tabulen zu erfinden, mit welchen man die vorgenändten sachen alle verrichten müchte” (Clark 2015, 58) as “to invent general tables with which you would like to do all of the above[mentioned] things” (121).

18 University Library Graz I 18600–18601.

19 In the usual case, where the number cannot directly be found in the table, Bürgi tells us to use linear interpolation (Clark 2015, 135–36). This might explain why the red numbers are listed in the table with an extra zero.

20 I provide a reason for the abstraction of the astronomical and trigonometrical context in this case in a forthcoming article.

21 Walton (2017, 90) uses logarithms as an exemplary case of theoretical mathematics that is distinct both from practical mathematical and application in material practice in early modernity.

22 While the surviving record of an astronomical calculation done by Bürgi seems to confirm the application of *prosthaphaeresis* in astronomy on first sight, the closer analysis I will present in a forthcoming article reveals that the seven digits he used for the sine values—the computation of which might indeed express the advantage of using *prosthaphaeresis* over traditional ways of multiplying by hand—went beyond any meaningful physical significance in their precision.

23 As mentioned earlier, the multiplication of sines frequently showed up in the computational practice of astronomers and implicitly also in the geometrical demonstrations of their algorithms. In this specific form, however, it seems to become an issue only with the attempt to give a proof of the method of *prosthaphaeresis*.

24 I deal with the political epistemology of Bürgi’s *Arithmetische und Geometrische Progress Tabulen* in a forthcoming article.

25 Clocks with second hands were extremely rare at this time, but Bürgi introduces it, among others, on an observational clock he constructed for Wiliam IV (Staudacher 2018, 127–31). Thanks to Samuel Gessner for this hint.

26 Its description was first published by Hulsius (1604).

27 A similar connection has been suggested by Schneider (2008, 66) in relation to Descartes’ conceptualization of the multiplication of line segments (see Mancosu 1992, 85).
